# A Gauze-Pad Wrapping of the Heart Can Save a Patient’s Life

**DOI:** 10.21470/1678-9741-2020-0589

**Published:** 2021

**Authors:** Manuel J. Antunes, João E. Bernardo, Carlos S. Pinto

**Affiliations:** 1 Department of Cardiothoracic Surgery, Faculty of Medicine, University of Coimbra, Coimbra, Portugal.

**Keywords:** Heart Ventricles, Rupture, Mitral Valve, Hemorrhage, Iatrogenic Diseases, Hemostatics

## Abstract

We describe one case of iatrogenic rupture of the left ventricle after mitral valve replacement and myectomy of the outflow tract. The cause and site of the rupture could not be identified, neither from the internal nor from the external examination. After unsuccessful use of hemostatic patches in the surface of the ruptured area, wrapping of the ventricles with a surgical gauze pad controlled the hemorrhage, hence saving the patient’s life.

**Table:** 

Abbreviations, acronyms & symbols	
AV	= Atrioventriculatr
CPB	= Cardiopulmonary bypass
HOCM	= Hypertrophic obstructive cardiomyopathy
LVOT	= Left ventricular outflow tract

Iatrogenic left ventricular rupture is a rare but well known and lethal complication of some cardiac procedures, with an incidence of 0.8-2.6%^[1,2]^. Rupture of the atrioventricular (AV) groove during mitral valve procedures, especially valve replacement, is the most frequent cause, but it can also be the consequence of too radical excision of papillary muscles or of erosion by bioprosthetic stents^[3]^. Rupture can also occur after myectomy of the left ventricular outflow tract (LVOT), but this involves mainly the septum, causing a ventricular septal defect, although free rupture can also happen^[4]^.

In any of these cases, the situation is critical and requires emergency surgical intervention, but the mortality remains very high as the rupture site is usually very fragile and frequently not amenable to repair. Mortality rates of 30-80% have been reported^[2,3]^. Several techniques have been described but none is universally applicable and a degree of inventiveness may be required in some cases^[5,6]^. We report a case where a previously undescribed approach was lifesaving.

A 79-year-old female patient was referred for surgery of severe mitral valve regurgitation caused by posterior leaflet (P2) prolapse. She also had hypertrophic obstructive cardiomyopathy (HOCM), particularly evident in the septum, with a LVOT of 45-50 mmHg. The aortic valve was normal.

HOCM was initially addressed by performing an extended myectomy. The mitral valve was treated by implantation of two pairs of artificial (polytetrafluoroethylene - PTFE) chordae in the prolapsing segment and of a 30 mm Carpentier-Edwards Physio® ring (Edwards). The valve became competent, but the intraoperative transesophageal echocardiogram showed obstructive systolic anterior motion with an LVOT gradient of 50 mmHg, which did not decrease with improved left ventricular filling. A decision was thus made to replace the mitral valve with a bioprosthesis (S. Jude Epic® 27 mm), with partial retention of the valve apparatus (posterior leaflet), using interrupted pledgeted sutures.

The patient was weaned from cardiopulmonary bypass (CPB) under a small-dose inotropic support. The immediate postoperative period was essentially uneventful. There was no significant early mediastinal or pericardial blood drainage, but on the second postoperative day there was a sudden surge of blood through the drains which persisted for four hours at a rate of approximately 250 ml/hour, with hemodynamic deterioration. There were no changes in the electrocardiogram.

A decision was made to reexplore the surgical field. Upon opening the pericardium and evacuation of clots, fresh red blood was seen coming from the back of the heart, but the origin could not be immediately ascertained. The patient was placed on CPB to facilitate manipulation of the heart. A large subepicardial hematoma was seen in the posterior wall of the left ventricle and a rupture of the AV groove was suspected.

The left atrium was opened, and nothing abnormal was seen. The mitral prosthesis was removed, and the AV grove was intact. The papillary muscles were not disrupted and the myectomy site did not appear suspicious of rupture. Another origin for the bleeding could not be identified from the inside. We could not differentiate whether this was a type 1 (AV groove) or type 2 (midventricular wall) rupture. The mitral prosthesis was reimplanted, and the atrium was closed.

On filling the heart, the hemorrhage from the posterior wall of the ventricle persisted. A patch of TachoSil® (human fibrinogen + thrombin, Baxter) was used to fill the subepicardial hematoma cavity and the surface was covered with Surgicel® (Ethicon), but it kept displacing into the pericardial well while the bleeding persisted. In despair, a double-layer ordinary surgical gauze pad was used to completely wrap the ventricular mass, which resulted in immediate cessation of the bleeding. It was finally decided to leave it in place for planned removal later.

The patient had an uneventful postoperative period. The systolic blood pressure was tightly controlled, not to exceed 100 mmHg, with continuous administration of sodium nitroprusside for 72 hours, after which the patient was extubated. There was minimal pericardial drainage during this period. As planned, the gauze pad was extracted on the 10^th^ postoperative day, during an uneventful re-sternotomy procedure, and there were no signs of bleeding from the back of the heart, but a full exploration was deliberately avoided. The decision and timing of removing the gauze were controversial, but it was thought that after 10 days the area of myocardial fragility would be reasonably consolidated. The patient was extubated on the table and had an uneventful recovery. She was discharged home on the 10^th^ day after the last procedure.

In conclusion, in our patient, an obvious cause and site of the rupture could not be identified, leaving a suspicious area in the posterior wall of the left ventricle. Hence, we had to resort to the use of anticoagulant/hemostatic patches which failed in adequately controlling the hemorrhage, especially because it became difficult to keep them in place. The idea of wrapping the ventricles with an ordinary surgical gauze came in despair and ended well. Hence, it was lifesaving in this particular case. And we know the case of a patient treated by a Brazilian colleague who used, with success, a commercially available super-glue (cyanoacrylate) for fixation of bovine pericardial patches to the epicardium (Lobo-Filho G: Personal communication).

As Mary Lou Cook, an American educator, said, “creativity is inventing, experimenting, growing, taking risks, breaking rules, making mistakes, and having fun”!

**Table:** 

Abbreviations, acronyms & symbols	
AV	= Atrioventriculatr
CPB	= Cardiopulmonary bypass
HOCM	= Hypertrophic obstructive cardiomyopathy
LVOT	= Left ventricular outflow tract
